# Supplementary material for: The Association between Vegan Dietary Patterns and Physical Activity—A Cross-Sectional Online Survey
Source: Nutrients. 2023 Apr 12;15(8):1847. doi: 10.3390/nu15081847 (PMC10145789; doi:10.3390/nu15081847)
Supplement: Supplementary file 1 [file nutrients-15-01847-s001.zip › nutrients-2326748-supplementary.pdf]

nutrients

MDPI

Supplementary Table S1: Population characteristics divided by dietary pattern based on modified PDI score

		Dietary patterns			
		**1st tertile Convenience** (n = 152)	**2nd tertile Traditional** (n = 172)	**3rd tertile Health-conscious** (n = 192)	*p*
Vegan since (years)		2.7 (2.5-3.0)	2.5 (2.5-3.0)	3.0 (3.0-4.0)	0.077
Age (years)		27.4 (6.0)	27.2 (7.0)	29.3 (9.3)	0.222
Gender	Female	78.8%	89.0%	86.5%	
	Male	16.6%	9.3%	12.5%	0.048
	Non-binary, not specified	4.6%	1.7%	1.0%	
Education	Compulsory school	9.2%	12.2%	5.2%	
	A-levels	44.7%	46.5%	46.4%	0.192
	University	46.1%	41.3%	48.4%	
Employment	Student or in training	37.5%	40.7%	43.2%	
	Employee or self-employed	57.9%	55.2%	56.3%	0.143
	Other	4.6%	4.1%	0.5%	
Smoking status	Yes	9.9%	7.6%	5.2%	
	Occasionally	12.5%	14.0%	8.3%	0.186
	Former	19.1%	15.1%	14.6%	
	Never	58.6%	63.4%	71.9%	
BMI (kg/m^2)		23.0 (4.4)	22.2 (3.4)	21.8 (3.2)	0.016
	Underweight	5.4%	2.9%	9.6%	
	Normalweight	75.8%	83.5%	77.1%	0.048
	Overweight	12.1%	10.6%	11.2%	
	Obesity	6.7%	2.9%	2.1%	

Metric data are given in mean (SD) or median (95% CI), if normal distribution was not given. Differences in groups were calculated using Chi-Square tests in categorical data, and ANOVA for metric data. P-values <0.05 were considered statistically significant.

Supplementary Table S2: Reasons for vegan diet & supplements divided by dietary patterns based on modified PDI score

		Dietary patterns			
		**1st tertile Convenience**	**2nd tertile Traditional**	**3rd tertile Health-conscious**	***p***
Reasons veganism*	Health aspects	52.3%	60.2%	64.9%	0.154
	Environmental protection	74.8%	73.7%	70.2%	
	Animal welfare	94.7%	91.8%	88.0%	
	Religious beliefs	1.3%	1.2%	0.5%	
	Weight reduction	1.3%	4.1%	3.7%	
	Taste	0.7%	0.6%	2.1%	
Supplements*	Vitamin B12	91.7%	93.8%	96.6%	0.056
	Vitamin D	72.9%	75.0%	76.0%	
	Omega-3-fatty acids	38.3%	42.5%	48.0%	
	Iodine	13.5%	18.8%	20.7%	
	Iron	45.1%	49.4%	48.0%	
	Calcium	14.3%	21.9%	20.7%	
	Zinc	23.3%	32.1%	37.4%	
	Potassium	9.0%	6.9%	8.9%	
	Vitamin K2	1.5%	3.1%	2.2%	
	Selenium	12.0%	25.0%	24.6%	
Vegan diet is varied#	Yes	82.2%	88.4%	96.4%	0.001
	No	6.6%	4.7%	2.1%	
	Not sure	11.2%	7.0%	1.6%	
Spending money due to vegan diet#	Spend more	36.2%	30.8%	21.1%	0.009
	Spend less	11.2%	11.0%	20.5%	
	No difference	47.4%	50.3%	48.4%	
	Not sure	5.3%	8.1%	10.0%	
Vegan friendly cafés/restaurants visits (Frequency)	Daily	2.0%	1.2%	0.0%	0.001
	Weekly	47.4%	37.8%	22.4%	
	Monthly	39.5%	42.4%	45.3%	
	2-6 months	10.5%	16.2%	29.2%	
	Never	0.7%	2.3%	3.1%	

*multiple responses; #personal opinion
Differences in groups were calculated using Chi-Square tests in categorical data and ANOVA for metric data. P-values <0.05 were considered statistically significant.

Supplementary Table S3: Physical activity divided by dietary patterns based on modified PDI score

	Dietary patterns			
	**1st tertile Convenience**	**2nd tertile Traditional**	**3rd tertile Health-conscious**	***p***
Sitting time (hours/week)	7.3 (3.3)	6.5 (3.1)	6.7 (2.7)	0.044
Aerobic PA (min/wk.)	240 (180-320)	270 (240-360)	360 (320-420)	0.006
Moderate (min/wk)	115 (90-120)	120 (120-180)	120 (120-165)	0.475
Vigorous (min/wk)	90 (60-120)	120 (120-180)	120 (120-180)	0.025
Strength training				
Never/<1 day/wk	45.3%	29.5%	26.3%	
1 day/wk	17.6%	21.1%	13.2%	<0.001
≥2 days/wk	37.2%	49.4%	60.5%	
Yoga (yes)	22.9%	32.8%	44.3%	0.014
Recommendations fulfilled				
Aerobic PA	67.4%	70.5%	79.1%	0.052
Strength training	37.2%	49.4%	60.5%	0.001
Aerobic PA + strength training	27.5%	36.5%	50.8%	<0.001

Metric data are given in mean (SD) or median (95% CI), if normal distribution was not given. Differences in groups were calculated using Chi-Square tests in categorical data and ANOVA for metric data. P-values <0.05 were considered statistically significant.

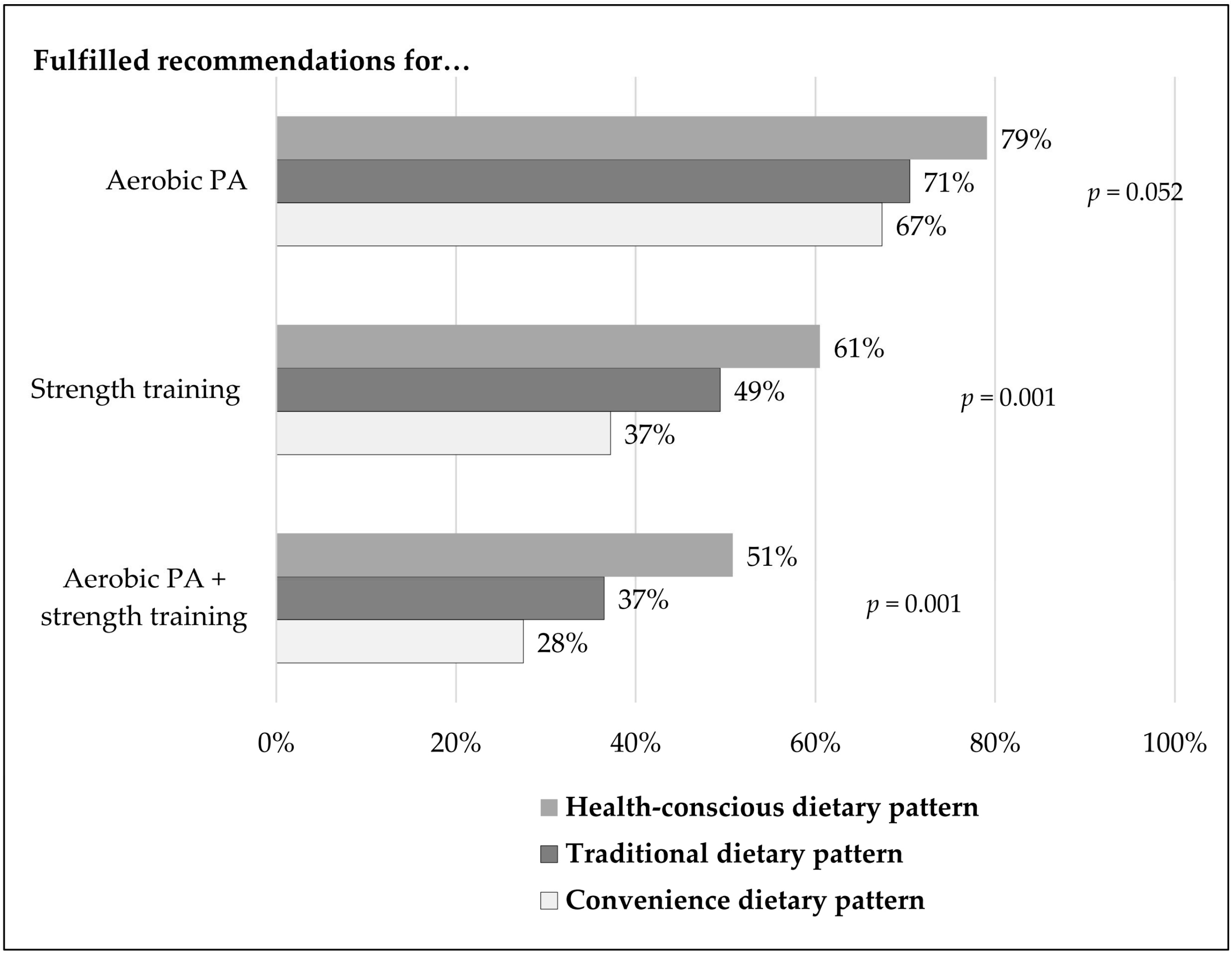

Supplementary Figure S1: Percentages of people fulfilling the physical activity recommendations. Dietary patterns are based on modified PDI score
